# Machine Learning Predictions on Outpatient No-Show Appointments in a Malaysia Major Tertiary Hospital

**DOI:** 10.21315/mjms2023.30.5.14

**Published:** 2023-10-30

**Authors:** Abdullah Fahim Ahmad Hamdan, Azuraliza Abu Bakar

**Affiliations:** 1 Pathology Department, Hospital Kuala Lumpur, Ministry of Health Malaysia, Kuala Lumpur, Malaysia; 2 Faculty of Information Science and Technology, Universiti Kebangsaan Malaysia, Selangor, Malaysia

**Keywords:** descriptive analysis, healthcare, machine learning techniques, no-show appointments, predictive analysis

## Abstract

**Introduction:**

A no-show appointment occurs when a patient does not attend a previously booked appointment. This situation can cause other problems, such as discontinuity of patient treatments as well as a waste of both human and financial resources. One of the latest approaches to address this issue is predicting no-shows using machine learning techniques. This study aims to propose a predictive analytical approach for developing a patient no-show appointment model in Hospital Kuala Lumpur (HKL) using machine learning algorithms.

**Methods:**

This study uses outpatient data from the HKL’s Patient Management System (SPP) throughout 2019. The final data set has 246,943 appointment records with 13 attributes used for both descriptive and predictive analyses. The predictive analysis was carried out using seven machine learning algorithms, namely, logistic regression (LR), decision tree (DT), k-near neighbours (k-NN), Naïve Bayes (NB), random forest (RF), gradient boosting (GB) and multilayer perceptron (MLP).

**Results:**

The descriptive analysis showed that the no-show rate was 28%, and attributes such as the month of the appointment and the gender of the patient seem to influence the possibility of a patient not showing up. Evaluation of the predictive model found that the GB model had the highest accuracy of 78%, F1 score of 0.76 and area under the curve (AUC) value of 0.65.

**Conclusion:**

The predictive model could be used to formulate intervention steps to reduce no-shows, improving patient care quality.

## Introduction

A ‘no-show’ appointment is when a patient fails to attend the scheduled appointment without prior notification to the healthcare provider. This issue remains one of the challenges for healthcare providers around the world. As the percentage of no-show incidents varies among healthcare centres, an absence or no-show during a clinical appointment can be costly and disruptive to the healthcare sector. When this happens, both sides will get affected: the patients and the healthcare providers. The patients will lose the opportunities to be treated for their medical condition and impact the patient’s health condition due to delayed diagnosis or treatment. Prospective patients might also be affected and less satisfied due to their inability to schedule timely appointments. Also, staff will be demoralised and unsatisfied, and the work process can become inefficient (1, 2).

Standard practice in healthcare centres is to allocate specific numbers of appointments for each operating day. An unused time slot due to no-shows can negatively affect the utilisation of space and resources. Additionally, this can increase the waiting time for consultation and hinder access to medical attention for those in better need (1, 2). The healthcare sector consistently struggles with increasing demand and cost pressure; healthcare organisations need to improve the effectiveness and efficiency of their services. Despite all the efforts taken, there are still several factors that lead to higher costs and underutilisation of resources. As described earlier, patient no-show appointments are one of the examples that match this issue.

Technological advances in information on healthcare and digitising health records have resulted in the rapid growth of the healthcare sector, including the usage of electronic medical record (EMR) systems by healthcare providers. The accumulation of data inside the EMR has given the vast opportunity to transform it into meaningful insights through big data techniques such as data mining, machine learning and predictive analysis. Predictive analysis has been used for other fields, such as the energy section, to generate electricity based on usage prediction. Another example includes weather forecast, where predictive analysis has become the core of weather analysis. Therefore, by analysing the EMR data and examining patient behaviour patterns, we could use big data analysis to predict a no-show occurrence.

In a typical healthcare setup, a patient is assigned an appointment date based on their diagnosis or medical condition. The appointment date is based on the available clinic/speciality slot. The healthcare provider schedules and prepare all the resources required for the appointment: the specialist/doctor slot, medical apparatus, medical records and other related resources. All these preparations usually take place days before the appointment date. This must be done to ensure the appointment session will happen most efficiently and conveniently to the patients and the staff themselves. The patient receives much-needed medical treatment, and the staff conducts their duties efficiently and in a productive environment (1, 2). Unfortunately, this does not happen in every appointment. A no-show or absence to the booked appointment will always occur, becoming an issue for the healthcare provider. The booked slot remains empty as the healthcare provider has received no prior notification. All time and effort spent to perform all the preparation are wasted. The patient cannot get the needed medical attention and care. Eventually, the patient may make more visits to the emergency department, where the treatments are much more expensive and there is less preventive care. This also hinders access to medical attention to those in better need, creating dissatisfaction among the patients and staff. This issue could become a severe problem for the healthcare provider if not overcome. The operational expenses are high, the workflow is inefficient and the care outcomes are suboptimal (3).

As resources are wasted, this translates into financial loss to the healthcare provider. In one study, the average no-show rate was as high as 18% and the cost per no-show patient was USD196 in 2008 (4). Another study explained that the US no-show rates are as high as 30% and those unfilled slots cost a physician USD200 on average (5). By considering these figures and accounting for the time and effort invested in each appointment and the potential discontinuation of care for the patient, we can grasp the significant financial impact of a no-show. Several interventions, such as sending reminder short message service (SMS) and phone calls, are useful in such issues (1–3, 6). However, this approach poses another issue. SMS and phone call imply additional costs to the healthcare provider; this approach is not cost-effective. The emergence of big data techniques and a better understanding of their application in the real world provides a vast opportunity for healthcare providers to change the accumulated patient data into new knowledge for better patient management. Studies (2, 6, 21) have shown that big data techniques like data mining, machine learning, and model prediction could solve the no-show problem.

However, limited research has been done on the appointment no-show occurrence in the Malaysian healthcare system and the impact of applying the machine learning approach to predict the patient no-show. Despite extensive research (10, 11, 12, 17, 22, 23) conducted globally, the lack of a similar process in Malaysian healthcare exhibits a gap in understanding the appointment no-show issue within a local environment. Therefore, this study proposes a predictive analytical approach to build a no-show appointment model in Hospital Kuala Lumpur (HKL) using machine learning algorithms.

### Related Work

The study by (7) was one of the first studies on predictive analysis for no-show appointments. They applied 20 predictors to a relatively small sample of 291 family practice centres. However, the results were unsatisfactory, as they only achieved 67.4% accuracy compared with the actual attendance rate of 73%. For this study, they applied linear discriminant combined with linear regression. A similar study was done by (8), which used a multivariate logistic regression technique to predict no-shows in a primary centre. Even though they did not report any performance indicators, they reported that the most significant features were age, race, the presence of any physician-identified psychosocial problems and the record of no-shows during the last 12 months.

Few studies that implemented regression models stand out for special attention. In (9), they reported a high AUC (area under the curve) value of 0.958 after implementing linear regression with L2 norm regularisation. They claimed that this high result contributed to the inclusion of features related to the patient’s diagnosis. Similarly, Alaeddini and Hong (10) adopted the idea of using penaliser feature selection. They proposed multinomial linear regression with L1/L2 regularisation and obtained close to 80% accuracy. However, they only used 410 appointments compared to 16,026 (9).

Conversely, a separate study indicated a higher accuracy of the decision tree model compared to linear regression (11). This accuracy value also surpassed the attendance rate. In another investigation, two algorithms rooted in information gain (JRip and Hoeffding) were employed to construct the decision tree (6). The historical record of no-shows, appointment location and specialty emerged as the most influential factors in the information-gain hierarchy. Both algorithms yielded respectable accuracies of 76.44% and 77.13%, along with AUC values of 0.776 and 0.861, respectively. However, both techniques fell short of achieving the attendance rate.

In a study, linear regression, Naïve Bayes (NB) and multilayer perceptron (MLP) models were implemented on a dataset comprising 73,811 appointment records (12). They found that the NB model best performed with an AUC of 0.86. Ensemble and stacking methods techniques combine predictions from multiple classifiers. In another study, gradient boosting was used to solve an imbalanced dataset, which produced an AUC value of 0.7404 (2). However, they concluded that the study needed more information about patients’ descriptions and appointment information for better prediction.

Younger adults, lower socioeconomic status, distance from the clinic, no private insurance, high waiting time and previous no-show history were found to be associated with no-show behaviour (13). Additionally, no universal variables can define the no-show problem as it depends heavily on the variables available in the EMR (14).

Feature selection is performed to enhance the prediction performance. The primary objectives of feature selection methods are to enhance prediction performance, produce faster and cost-efficient predictors and enable a better understanding of the data. This technique can be divided into three groups: i) filter, ii) wrapper and iii) embedded (15). Decision trees and penalised regression are commonly used for feature selection. In addition, the studies that applied penalised linear regression presented the best results (14).

Based on the publication distribution by year and the size of the datasets, most of the studies were done in the last decade. This shows the current interest in the no-show prediction problem. Additionally, there was an increasing size of the dataset used, which the recent availability of EMR can explain. The same study also found that from 50 studies reviewed, regression models were mainly used (30 studies). Other models were tree-based, neural networks, Markov-based, Bayesians and ensemble/stacking models. Some of the studies applied multiple predictive models (14).

Currently, there is a scarcity of research addressing the no-show issue in Malaysia. One study examined the no-show rate within a diet clinic at Hospital Sultan Ismail (HSI) and employed SMS interventions to mitigate this problem (16). Impressively, they decreased the no-show rate from 40.7% to 22.2%. Nonetheless, it is important to note that this study utilised a limited dataset encompassing no more than 170 patients. Additionally, the financial implications of SMS utilisation were particularly pronounced for healthcare providers operating within larger institutions serving a more extensive population.

## Methods

### Experiment Setup

**Figure 1:**
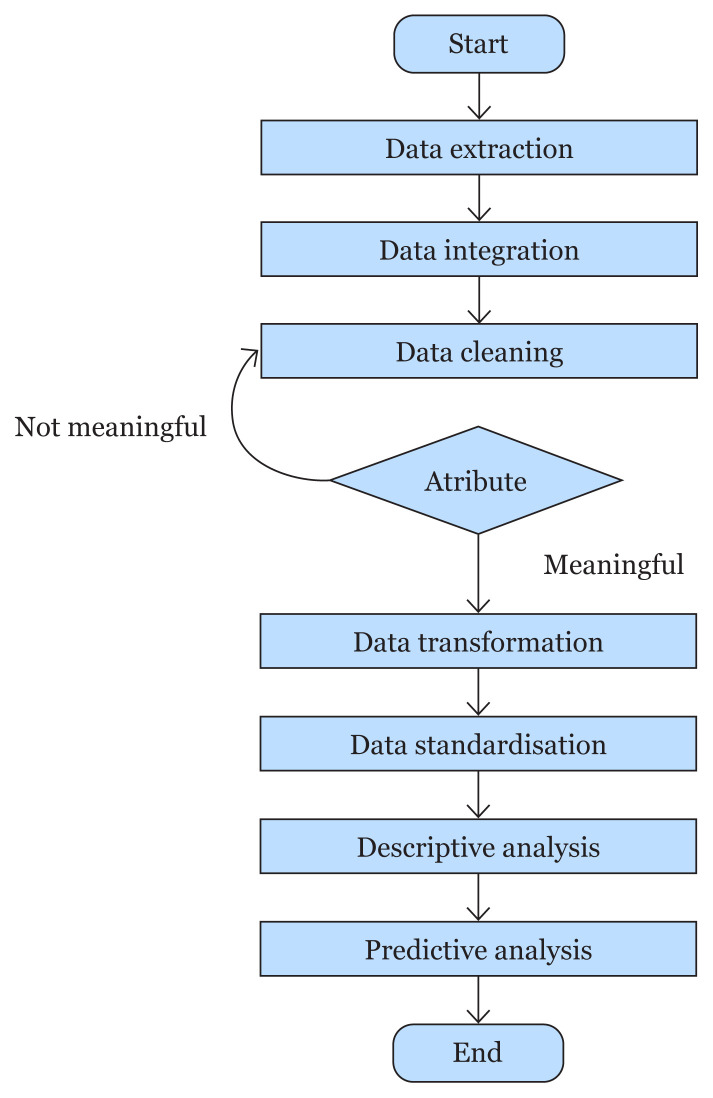
Workflow of research methodology

This study is based on Cross-Industry Standard Process for Data Mining (CRISP-DM) and consists of seven main steps, illustrated in Figure 1. The first step is extracting the patient appointment data from the HKL’s patient management system (SPP). The outpatient data from 2019 was used in this study, excluding data from Paediatrics and Obstetrics & Gynaecology (O&G) clinics. Then, these data, consisting of three primary tables (appointment, encounter and person), underwent a data integration process to generate a single dataset. Data cleaning and transformation are performed to handle all the missing data, duplicates and inconsistent or incomplete data.

Data standardisation brings all the data in a uniform format to enhance the modelling performance and eliminate possible bias. Descriptive analysis was done by visually plotting the data to explore the relationship between each attribute and the no-show appointments. Finally, predictive models were constructed using seven machine learning algorithms for the no-show prediction. The algorithms used were logistic regression (LR), decision tree (DT), k-near neighbours (kNN), NB, random forest (RF), gradient boosting (GB) and MLP. Lastly, decision rules were generated by the DT model. The model used the DT algorithm in this phase to produce decision rules based on the attributes and outcomes (show or no-show). Subsequently, these rules were presented to the experts from HKL for feedback. These experts were officers involved in the management of patient appointments in their respective clinics and had a minimum of 5 years of experience.

Three different train/test splits (60:40, 70:30 and 80:20) with stratified sampling were used for the predictive modelling. Ten-fold validation was done on each split to ensure the classifier saw all training data and minimised the error. The evaluation metrics, consisting of accuracy, AUC value and F1 score, were used to gauge the performance of each model. These experiments are done using Python on an Intel Core i5 2.90 GHz CPU with 16 GB RAM.

### Dataset

A final dataset of 246,943 appointment data with 14 attributes was used for the descriptive and predictive analysis. The dataset consists of patient demography (gender, age and state of residence) and appointment data (clinic-referred, appointment booking details, actual appointment details and no-show records). This data refined appointment booking details and generated new variables (*created_date_D, created_date_M* and *created_date_Y*). Additionally, actual appointment details were refined to the day and month of the appointment (*reserve_weekday* and *reserve_month*). The timespan between the booking date and the actual appointment date was counted and put as *waiting_days* attributes. The full description of the dataset is shown in Table 1.

## Results

### Descriptive Analysis

The ‘no-show*’* attribute, the target class, categorises the dataset into show and no-show appointments. Based on the analysis, 69,173 patients did not attend appointments, accounting for 28% of the dataset. Based on the literature review, this figure did not vary so much from studies in neighbouring countries (17). Nevertheless, this value is still high as various other issues can arise from this absenteeism problem and require efforts to address the problem of non-compliance with these appointments.

**Figure 2:**
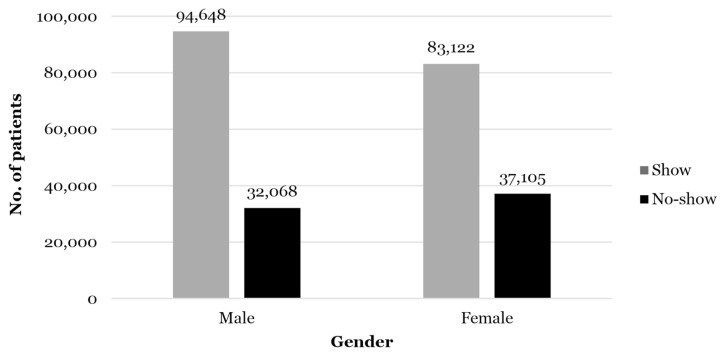
No-show appointments based on gender

The descriptive analysis also found that most appointments in 2019 accounted for male patients, with this group exceeding female patients by 6%, as shown in Figure 2. In contrast, no-show appointments among female patients were 7% more than male patients. This correlation may be due to these female patients having family commitments, dependence on their partner or other factors. However, these factors were not studied in this study. In addition, the median age of patients in the data set of this study was 56 years old, with patients aged 57 years old–67 years old dominating the patient distribution. As expected, the distribution of no-show appointments was also according to the age distribution of the patients.

The three clinics with the most appointments were the ophthalmology, orthopaedic and medical outpatient department (MOPD). However, some clinics have no-show appointments that exceed or are almost equal to the number of patients present, namely urology, nephrology, oncology and radiotherapy clinic, with no-show rates of 58%, 51%, and 50%, respectively. Looking at the type of appointment, most of the appointments are recurring appointments (90%). However, there was no significant difference in the number of no-show appointments by appointment type. Both types of appointments (new and recurring) showed the same percentage of 40% and 39%, respectively.

The HKL provides outpatient services to those who live in the Federal Territory of Kuala Lumpur and Selangor. Patients from these two states constitute 60% of the total patients in this data set. However, many patients do not have a form of residence information in this dataset. This proportion of information was purposely not eliminated during the data cleaning process as it also holds other information that can be used for modelling. Ultimately, as HKL is the country’s main referral hospital, residence information is vital to devise intervention measures to address the problem of non-attendance of these appointments.

**Figure 3:**
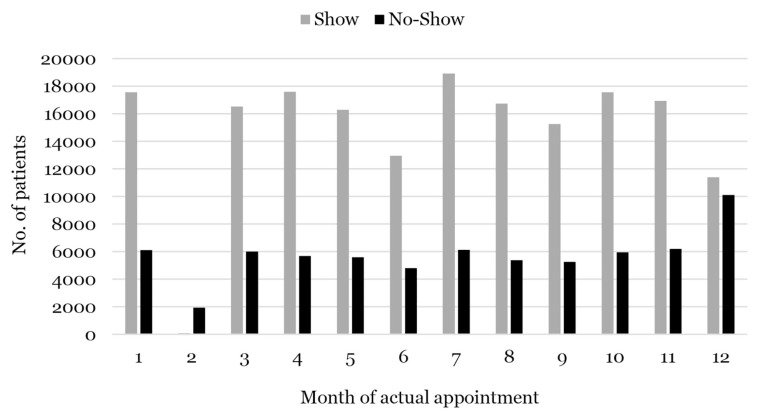
No-show appointments based on month

Two new attributes were created for the appointment date: appointment day (*reserve_weekday*) and time interval period (*waiting_days*). According to the literature review, these two attributes influence the absence of appointments. The highest time interval is 798 days which is more than 2 years compared to the shortest time, 1 day. The variability of this time interval may be due to this data set involving various disciplines of expertise, the number of specialists/physicians, the complexity of the disease and other various factors. In addition, no specific day is the patient’s choice for an appointment because the distribution of attendance and absence was the same every day except Friday. Friday had the lowest total attendance due to shorter operating hours than other days. The appointment month factor also plays an important role, with the highest percentage of no-shows recorded in February and December, as shown in Figure 3. This trend is believed to occur due to the existence of long public holidays such as festive holidays and school holidays.

### Predictive Analysis

Twenty-one models were developed using seven machine learning algorithms and three distinct data splits. Table 2 describes all the model performances based on the evaluation matrix. Accuracy is a common metric used in classification problems as it is easy to calculate and compare. In their research, the accuracy of most of the no-show models was between 67.4% and 91.11% (14). Meanwhile, a study found that their no-show model had an accuracy of 76.5% (6).

Meanwhile, Dantas et al. (18) reported an accuracy of 71%. In this study, the GB model showed the highest accuracy of 78%. While the model demonstrated accuracy levels comparable to those reported in prior studies, it’s essential to recognise that relying solely on accuracy for performance comparison may not be comprehensive enough. This is because the prevalence of the majority class can easily influence accuracy. Other evaluation matrices such as recall, precision, F1 score and AUC were more suitable for classification problems with an imbalanced dataset (19).

**Figure 4:**
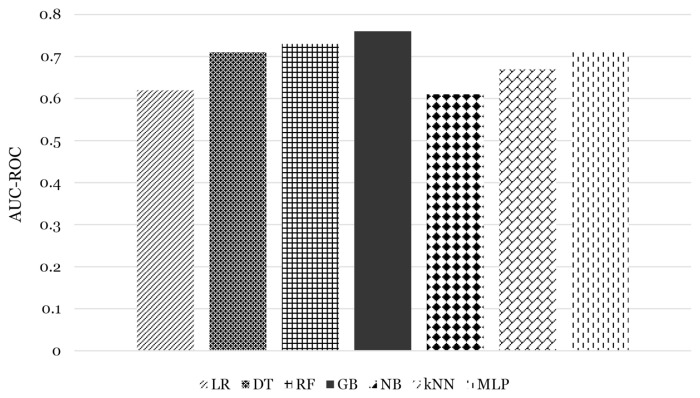
Comparison of AUC-ROC value between the models

Recall or sensitivity explains how well our models predict the true positive (no-show appointments) against the actual no-show appointments. Recall values for all the models were between 0.72 and 0.78, with the GB model having the highest value. In their study, Gromisch et al. (20) reported 0.75 for the sensitivity of their model. On the other hand, the models showed precision values of 0.68–0.77, with the GB model again showing the highest value. Based on the previous studies, Lee at al. (21) reported a precision of 0.79 for their GB model; meanwhile, the LR model developed by Kurasawa et al. (9) had a precision of 0.76. They also used the F1 score to evaluate their model and reported a score of 0.70. The F1 score is a harmonic mean between recall and precision. Therefore, it is not easily affected by outliers in both values. Model GB showed the highest F1 score (0.76) while the LR model scored 0.63 (lowest score). Area under the ROC curve (AUC-ROC) was selected for performance evaluation as it is unaffected by the majority and minority class imbalance. As illustrated in Figure 4, the GB model has the highest AUC-ROC value (0.76) and the LR model has the lowest value (0.62). The GB model showed the highest value for all the evaluation matrices.

### Expert Evaluation

The evaluation of the decision rules was made by four officers directly engaged in the management of patient appointments. Based on Table 3, various responses were received from officers managing patient appointments. Only 7 (19%) out of 36 officers agreed on the rules, 18 responses (50%) were unsure and the rest disagreed. As described earlier, the no-show appointments issue at HKL has never been studied. The interviews with the officers acknowledged the problem of appointment non-attendance. However, the case could not be studied in detail due to human resource constraints and the increased number of patients yearly.

In addition, the lack of data or basic studies and the analytical forecasting approach regarding the no-show appointments is still new to them. These officials are not able to provide accurate answers to all the rules. Also, the officers offered several important factors or attributes that could contribute to the issue of no-shows; the data were unavailable in the SPP. These factors are the change in the appointment date, the type of transportation to the appointment (own vehicle, public transport, sent child/guardian or so on) and the financial level of the family (such as M40 and B40).

## Discussion

A comparison of model performance in this paper with existing studies is presented in Table 4. It should be noted that some of the current studies performed better than the models in this paper. This is because imbalanced datasets are common in the real world, especially in healthcare. Most supervised learners tend to classify by prioritising the majority class and overlooking the minority class. In this situation, relying solely on model accuracy is inaccurate, as it could lead to wrong model selection. Therefore, the model performance must be done using additional evaluators such as recall, precision, F1 score and AUC-ROC values. Based on Table 2, the GB model was selected as the best model in this study, showing the highest value in the evaluation matrix. GB is an ensemble learner and is not easily affected by the imbalance of class distribution in the dataset. The GB algorithms have also performed well using discrete and continuous data (2). Meanwhile, the data split at 70:30 gave the models better performance in the evaluation.

Even though LR is the most common algorithm used in most of the no-show research, the LR model in this study exhibited a slightly low AUC-ROC value compared to the other research. This might be due to the feature selection techniques used by the different research. For instance, the study by Alaeddini and Hong (10) uses the LR penaliser and L1/L2 regularisation techniques, which resulted in model accuracy up to 80%. Meanwhile, Harvey et al. (22) used stepwise LR and (17) multiple LR, which can have better AUC-ROC results for their models.

The type of data used also plays a significant role in modelling. Previous research proved that a patient’s clinical history data and socioeconomic and educational background can better predict no-show appointments (14). This study found that the number of attributes for the DT model resembled research by AlMuhaideb et al. (6) and Praveena et al. (11). However, these studies reported higher accuracy by using patients’ clinical data. A comparison between the NB model and previous research by Mohammadi et al. (12) and Topuz et al. (23) also showed the same occurrence. These studies used employment status, insurance data, income level and medical history data, and reported higher AUC-ROC values.

Additionally, variations in model performances obtained by this study were also due to an imbalance in class distribution, dataset size, feature selection technique, type of data or attributes used in modelling and the machine’s natural learning algorithm. Nevertheless, the model’s performance in this study is comparable to existing studies.

### Proposed Intervention Strategies

Based on the decision rules generated, these intervention strategies are proposed to reduce no-show appointments:

- The recommended interval time for patients less than 80 years old of age from the surgical outpatient department (SOPD) clinic, Dietetics Unit, Oncology and Urology Day Care Unit is less than 13 days or more than 57 days. This situation is for appointments scheduled during January and November only.
- For January–July, avoid booking Wednesday as an appointment day. This condition is valid for the October and November appointment month and an interval period of fewer than 107 days.
- For patients over the age of 59 years old and from clinics other than Anaesthesiology, Endoscopy Daycare, Genetics Daycare, Geriatric Daycare, Medical Daycare, Surgical Daycare, Genetics Clinic, MOPD Clinic and Nephrology Clinic, the suggested appointment days are Tuesday as well as Thursday. This condition is valid for appointments from January to November and has an interval of over 58 days.

## Conclusion

No-show appointments are a problem that needs to be resolved. It can lead to potential problems, such as disruption to patient treatment continuity, higher healthcare provider operating costs and resource wastage. Various studies have suggested that a predictive analytical approach using machine learning can help solve the no-show appointment issue.

This study showed that the GB model performs best in predicting no-show appointments using real patient data from HKL, with an accuracy of 78%, recall of 0.77, F1 score of 0.76 and AUC value of 0.76. This can be attributed to the fact that the GB algorithm is an ensemble classifier and a better classifier in imbalanced datasets. Differences in AUC value for the LR model were due to different feature selection techniques applied in the modelling process. Although this study uses a similar number of attributes to the existing studies for the DT model, the difference in accuracy values obtained is driven by differences in the data types or attributes used. The performance differences of the NB model are also due to the same factors.

This study could be expanded using additional patient data, such as SPP patient data from 2015 to 2020. Patient-related data unavailable in the SPP are occupational background, socioeconomic and clinical data, such as disease diagnosis can be obtained to get a more accurate predictive model. In addition, hyperparameter optimisation is also proposed to enhance the accuracy of the prediction model.

## Figures and Tables

**Table 1:** Description of the dataset

No.	Attributes	Data Type	Description
1	*machine_location*	Categorical	Clinic referred
2	*gender_code*	Binary	Patient’s gender (Male/Female)
3	*person_cur_state*	Integer	Patient’s state of resident
4	*person_age*	Integer	Patient’s age
5	*no_show*	Binary	No-show (‘0’: No; ‘1’: Yes)
6	*reserve_weekday*	Nominal	Day of the actual appointment
7	*reserve_month*	Nominal	The month of the actual appointment
8	*waiting_days*	Integer	Days between the date of appointment booking and the actual appointment
9	*reserve_date_Y*	Integer	Year of the actual appointment
10	*reserve_date_M*	Integer	The month of the actual appointment
11	*reserve_date_D*	Integer	Date of the actual appointment
12	*created_date_Y*	Integer	Year of appointment booking
13	*created_date_M*	Integer	The month of appointment booking
14	*created_date_D*	Integer	Date of appointment booking

**Table 2:** Performance of each model

Model	Data splits	Accuracy	Recall	Precision	F1 score	AUC
LR	80:20	72	0.72	0.68	0.63	0.62
	70:30	72	0.72	0.68	0.63	0.62
	60:40	72	0.72	0.68	0.63	0.62
DT	80:20	75	0.75	0.73	0.73	0.71
	70:30	75	0.75	0.73	0.74	0.71
	60:40	75	0.75	0.73	0.73	0.70
RF	80:20	76	0.76	0.76	0.71	0.73
	70:30	76	0.76	0.76	0.72	0.73
	60:40	76	0.76	0.76	0.71	0.73
GB	80:20	**78**	**0.78**	**0.77**	**0.76**	**0.76**
	70:30	**78**	**0.78**	**0.77**	**0.76**	**0.76**
	60:40	**78**	**0.78**	**0.77**	**0.75**	**0.75**
NB	80:20	72	0.72	0.66	0.63	0.61
	70:30	72	0.72	0.66	0.62	0.61
	60:40	72	0.72	0.66	0.62	0.61
kNN	80:20	74	0.74	0.71	0.70	0.67
	70:30	74	0.74	0.71	0.70	0.67
	60:40	71	0.74	0.71	0.69	0.66
MLP	80:20	76	0.76	0.75	0.70	0.62
	70:30	75	0.75	0.74	0.71	0.71
	60:40	76	0.75	0.74	0.70	0.70

Note: Bolded readings indicated the results from the best model and the highest values among the evaluation metrics.

**Table 3:** Decision rules generated using the DT model

No.	Decision rules	Expert Evaluation (Agree/Not sure/Not agree)
1	(*machine_location* ≤ 28.5) AND (*reserve_date_M* ≤ 11.5) AND (*waiting_days* ≤ 57.5) AND (*waiting_days* ≥ 12.5) AND (person_age ≤ 80.5) = no-show	1 Agree 2 Not sure 1 Not agree
2	(*reserve_date_M* ≥ 9.5) AND (*reserve_date_M* ≤ 11.5) AND (*waiting_days* ≤ 107.5) AND (*created_date_M* ≤ 7.5) AND (*reserve_weekday* ≥ 5) = no-show	2 Not sure 2 Not agree
3	(*machine_location* ≤ 9.5) AND (*reserve_date_M* ≤ 2.5) AND (*waiting_days* ≤ 169.5) AND (*waiting_month* ≥ 3.5) = no-show	1 Agree 3 Not sure
4	(*reserve_date_M* ≤ 11.5) AND (*waiting_days* ≥ 57.5) AND (*machine_location* > 12) AND (person_age ≥ 58.5) AND (*reserve_weekday* ≤ 3.5) = no-show	1 Agree 2 Not sure 1 Not agree
5	(*reserve_date_M* ≥ 11.5) AND (*reserve_date_D* ≤ 17.5) AND (*machine_location* ≤ 17.5) AND (*created_date_M* ≤ 10.5) AND (*waiting_days* ≤ 142) AND (*person_cur_state* ≤ 4.5) = no-show	2 Not sure 2 Not agree
6	(*machine_location* ≥ 28.5) AND (*reserve_date_M* ≤ 3.5) AND (*reserve_month* ≤ 3.5) AND (*person_age* ≤ 77.5) = no-show	2 Not sure 2 Not agree
7	(*machine_location* ≥ 28.5) AND (*reserve_date_M* ≥ 3.5) AND (*gender_code* ≤ 0.5) AND ((*reserve_month* ≤ 3.5) AND (*waiting_days* ≤ 49.5) AND (*created_date_M* ≤ 77.5) = no-show	1 Agree 2 Not sure 1 Not agree
8	(*machine_location* ≥ 28.5) AND (*reserve_date_M* ≥ 3.5) AND (*gender_code* ≤ 0.5) AND (*person_age* ≥ 61.5) AND (*waiting_days* ≤ 67.5) AND (*person_cur_state* ≤ 12.0) = no-show	2 Agree 1 Not sure 1 Not agree
9	(*machine_location* ≥ 28.5) AND (*reserve_date_M* ≥ 3.5) AND (*gender_code* ≤ 0.5) AND (*person_age* ≥ 61.5) AND (*waiting_days* ≥ 67.5) AND (*person_cur_state* ≤ 8.5) AND (*reserve_month* ≤ 3.5) = no-show	1 Agree 2 Not sure 1 Not agree

**Table 4:** Model performance of no-show appointments prediction

Model	Authors	Study location	No. of patients	No. of appointments	No-show (%)	No. of attributes	Model performance
GB	Lee et al. (21)	KK Women’s and Children’s Hospital, Singapore	-	1 million	25.4	42	0.832 (AUC)
	Elvira et al. (2)	San Carlos Clinical Hospital, Madrid	323,664	2,234,119	10.6	28	0.74 (AUC)
	This research	HKL, Malaysia	246,943	246,943	28	13	0.76 (AUC)
LR	Alaeddini and Hong (10)	Veteran Affairs (VA) Medical Center, USA	–	410	–	9	80 (ACC)
	Harvey et al. (22)	Massachusetts General Hospital	–	54,652	6.5	27	0.753 (AUC)
	Chua and Chow (17)	Changi General Hospital, Singapore	–	75,677	28.6	14	0.72 (AUC)
	This research	HKL, Malaysia	246,943	246,943	28	13	0.62 (AUC) 72(ACC)
DT	AlMuhaideb et al. (6)	King Faisal Specialist Hospital and Research Centre Organization (KFSHRC), Saudi Arabia	–	1,087,979	11.3	11	76.5 (ACC)
	Praveena et al. (11)	Brazilian dataset	–	100,000	20	12	89.6 (ACC)
	This research	HKL, Malaysia	246,943	246,943	28	13	0.71 (AUC) 75 (ACC)
NB	Mohammadi et al. (12)	Community health centres in USA	73,811	73,811	16.7	18	0.86 (AUC)
	Topuz et al. (23)	Kansas University School of Medicine	16,345	105,343	–	10	0.691 (AUC)
	This research	HKL, Malaysia	246,943	246,943	28	13	0.61 (AUC) 72 (ACC)
